# High-Quality GaSe Single Crystal Grown by the Bridgman Method

**DOI:** 10.3390/ma11020186

**Published:** 2018-01-24

**Authors:** Tao Wang, Jie Li, Qinghua Zhao, Ziang Yin, Yinghan Zhang, Bingqi Chen, Yong Xie, Wanqi Jie

**Affiliations:** 1 State Key Laboratory of Solidification Processing, Northwestern Polytechnical University, Xi’an 710072, China; lijiejane333@mail.nwpu.edu.cn (J.L.); nwpugate@mail.nwpu.edu.cn (Q.Z.); yindy90@mail.nwpu.edu.cn (Z.Y.); 15591809327@163.com (Y.Z.); chenbq@mail.nwpu.edu.cn (B.C.); jwq@nwpu.edu.cn (W.J.); 2 Key Laboratory of Radiation Detection Materials and Devices, Ministry of Industry and Information Technology, Northwestern Polytechnical University, Xi’an 710072, China; 3 State Key Discipline Laboratory of Wide Band Gap Semiconductor Technology, School of Advanced Materials and Nanotechnology, Xidian University, Xi’an 710071, China; yxie@xidian.edu.cn

**Keywords:** ε-GaSe, crystalline quality, two-dimensional materials, photodetector

## Abstract

A high-quality GaSe single crystal was grown by the Bridgman method. The X-ray rocking curve for the studied GaSe sample is symmetric and the Full Width at Half Maximum (FWHM) is only 46 arcs, which is the smallest value ever reported for GaSe crystals. The IR-transmittance is about 66% in the range from 500 to 4000 cm^−1^. The photoluminescence spectrum at 9.2 K shows a symmetric and sharp excition peak in 2.1046 eV. The results indicate that the as-grown GaSe crystal is of high crystalline quality. The as-grown ε-GaSe crystal has a p-type conductance with the resistivity of 10^3^ Ω/cm, and the Hall mobility is ~25 cm^2^ V^−1^ s^−1^. Few-layer GaSe crystals were prepared through mechanical exfoliation from this high-quality crystal sample. Few-layer GaSe-based photodetectors were fabricated, which exhibit an on/off ratio of 10^4^, a field-effect differential mobility of 0.4 cm^2^ V^−1^ s^−1^, and have a fast response time less than 60 ms under light illumination.

## 1. Introduction

GaSe is known as a non-linear optical material, which possesses great potential in non-linear optical devices and THz generation [1,2,3,4,5,6,7,8]. Recently, two-dimensional (2D) GaSe materials have drawn the increasing attention of researchers because of their superior performance in optical and electrical aspects. Hu et al. [9] reported a photodetector based on few-layer GaSe materials for the first time, with an responsivity of 2.8 A/W under 254 nm illumination and a corresponding quantum efficiency of 1367%, which is much higher than MoS_2_ and other two-dimensional semiconductor materials, such as grapheme. Since then, many efforts have been made to improve the performance of 2D GaSe devices through changing the device structure or device processing [10,11,12]. Meanwhile, obtaining high-quality 2D GaSe materials is also an important way to improve device performance. 

2D GaSe is often prepared by mechanical exfoliation from bulk GaSe [13,14,15] or grown by vapor phase transport techniques [16,17,18,19,20,21]. Lei et al. [16] reported vapor phase transport method to grow GaSe atomic layers for the first time, and the photoconductivity measurements shows an on/off ratio of 10^3^. GaSe nanoplates epitaxial growth on transparent flexible mica substrates showed photo-responsivity of 0.6 A/W [17]. Recently, Xiong et al. [19] succeed in synthesizing GaSe nanoribbons through one step thermal deposition process. The GaSe nanoribbon-based photodetectors showed an on/off ratio of 10^3^, a field-effect differential mobility of 0.03 cm^2^ V^−1^ s^−1^, and the response time is less than 0.3 s. In general, those methods use Ga/Ga_2_Se_3_ or Ga_2_Se_3_ as source materials, which may result in heterogeneous or non-stoichiometric composition and morphology. These introduced impurities and defects will finally deteriorate the performance of devices. Mechanical exfoliation is a promising method where the high quality can be inherited from the bulk crystal and large scale 2D GaSe can be obtained. However, most GaSe materials used to fabricate 2D GaSe devices through mechanical exfoliation were bought from commercial vendors, making it difficult to fully understand the properties of the materials. There have been many attempts to grow GaSe crystals [20,21,22,23,24,25,26,27], but the growth of high-quality bulk GaSe crystal is still a challenge. The great difference in saturation vapor pressure of the components Se and Ga can cause non-stoichiometry of a melt-grown crystal composition. Another obstacle is the dendrite growth caused by instability of the growth front [28]. The X-ray rocking curve and IR-transmittance results reveals that crystalline quality still needs to be improved. In addition, high-quality crystals are also required to reduce scattering and improve the phase matching for nonlinear applications. 

In this work, we report our efforts on the grown of high-quality GaSe crystals by the Bridgman method. Great crystalline quality was exhibited by several methods. 2D GaSe devices were fabricated based on the high-quality crystal. The few-layered GaSe photodetector exhibits a high on/off ratio of 10^4^, a field-effect differential mobility of 0.4 cm^2^ V^−1^ s^−1^, and has a response time less than 60 ms under light illumination.

## 2. Experimental Section

### 2.1. Synthesis of GaSe Polycrystals

High-purity Ga (6N, 99.9999%) and Se (6N, 99.9999%) from Emei Corp., Ltd. (Emei, China) was used as the raw material. The fused quartz ampoule coated with a carbon film was used to prevent adhesions. Gallium was baked at 673 K for 4 h under high vacuum to remove the oxide layer. Ga and Se were mixed in a stoichiometric ratio and sealed at 10^−5^ Torr. A single-temperature zone rocking furnace was used for synthesis. 

### 2.2. Growth of Single-Crystal GaSe

A two-temperature zone furnace was used to grow the GaSe single crystal by the vertical Bridgman method. The upper and lower zones were set at 1293 K and 1173 K, separately, and the temperature gradient was 10 K/cm. The crystal growth rate was 0.5 mm/h. A BN-crucible with an inner diameter of 22 mm was used to grow the GaSe crystal.

### 2.3. Characterization Methods

The phase and crystal structure of the as-grown GaSe crystals were identified by powder X-ray diffraction. The rocking curve was obtained from (004) reflex of the cleaved GaSe face using an Empyrean X-ray diffraction machine (PANalytical, X’Pert Pro, Eindhoven, The Netherlands). The morphology was characterized by scanning electron microscopy (SEM) using a Supra-55 (Carl Zeiss, Jena, Germany). The composition of GaSe was measured by an electro-probe microanalyzer (EPMA; JXA-8100, JEOL, Tokyo, Japan). The infrared transmittance from 500 to 4000 cm^−1^ was measured by a Nicolet Nexus Fourier Transform Infrared Spectrometer (Nexus 670, Nicolet, Waltham, MA, USA). The optical energy gap at room temperature was determined by a UV-3150 ultraviolet visible/near-infrared spectrometer (Shimadzu, Kyoto, Japan). The photoluminescence spectrum was measured by using an argon ion laser with the wavelength of 488 nm and the luminescence signals were recorded with the spectrometer of TRIAX 550 (Jobin Yvon, Paris, France). The I–V characteristic curve was measured with an Agilent 4155C semiconductor parameter analyzer (Agilent, Santa Clara, CA, USA). The Hall measurement was carried out on Keithley HMS Model 7077 (Tektronix, Beaverton, OR, USA) to identify the carrier type and concentration, and to obtain the mobility of the sample in Van der Pauw geometry.

### 2.4. The Preparation of GaSe (Metal-Oxide Semiconductor Field-Effect Transistor) MOSFET Photodetector

Few-layered GaSe was obtained by mechanical exfoliation by using scotch tape. Photodetectors were fabricated on p-doped Si substrates with a 300 nm SiO_2_. A Ti (5 nm)/Au (50 nm) electrode was deposited on clean SiO_2_/Si substrates by magnetron sputtering assisted with a mask. Few-layered GaSe was transferred onto the electrodes using poly (dimethyl siloxane) (PDMS). The devices were annealed under high vacuum for 2 h at 200 °C to reduce contact resistance. Electrical measurements were made by using an Agilent 4155C (Agilent, Santa Clara, CA, USA) combined with a probe station.

## 3. Results and Discussion

### 3.1. Crystalline Structure and Composition

**Figure 1:**
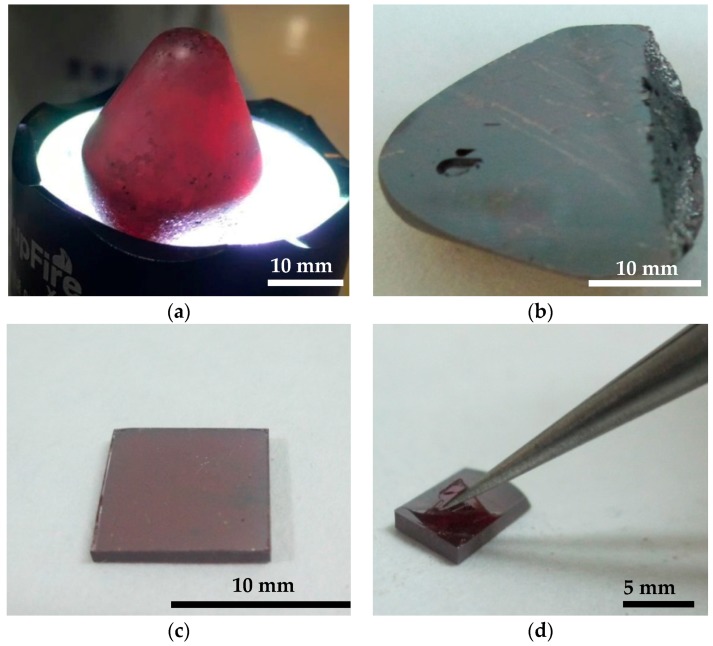
(**a**) The as grown GaSe crystal under light; (**b**) the GaSe crystal cleaved along the (001) face; (**c**) the GaSe single-crystal wafer of 10 × 10 × 1 mm; and (**d**) the cleaved surface of GaSe wafer.

A GaSe ingot with diameter of 22 mm and length of 20 mm was obtained, as shown in Figure 1a. The crystal is transparent and shows rufous color with a light illumination. Figure 1b shows the sample cleaved along the (001) layers from as-grown ingots with no additional treatment or polishing of the surface. The whole surface indicates one large grain along the cleavage surface, and several voids are seen at the surface. Figure 1c shows a GaSe single-crystal wafer of 10 × 10 × 1 mm and Figure 1d indicates that it can be easily exfoliated because of the weak interlayer van der Waals interaction. The SEM pattern of the cleavage surface (see supplementary materials Figure S1) shows the laminated structure and it is atomically flat without any micro-cracks or defects.

**Figure 2:**
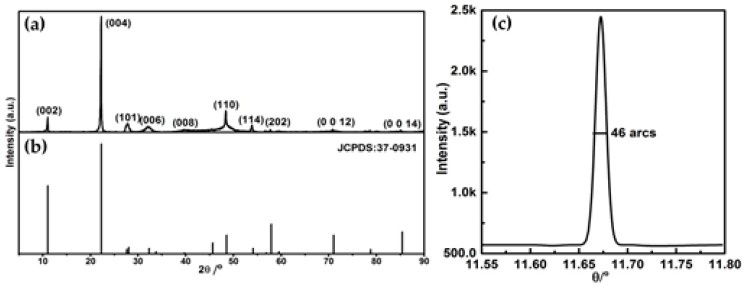
(**a**) The powder X-ray diffraction pattern (GaSe) of GaSe sample; (**b**) the PDF database of GaSe (JCPDS: 37-0931); and (**c**) the rocking curve of the (004) face.

Figure 2a,b shows the powder diffraction pattern of the as-grown GaSe crystal and the theoretical curve of GaSe (JCPDS: 37-0931). They are in good agreement and confirm that hexagonal GaSe crystal is grown with cell parameters a = b = 3.749 Å, and c = 15.907 Å, space group D3h1 (P$\bar{6}$m2).

Usually, the symmetry of the X-ray rocking curve is directly related to the structural uniformity of the crystal. Figure 2c shows the X-ray rocking curve of the (004) face of the as grown GaSe crystal. The peak shape is symmetric and the FWHM is about 46 arcs, which is the smallest values ever reported for GaSe crystals (0.15° in [20], split peaks in [21], 0.07° in [24], and 0.04° in [28]). The results indicated the as-grown GaSe crystals has a good crystalline quality.

Table 1 shows the composition distribution from the tip to tail of the GaSe ingot, as measured by EPMA. The Ga/Se ratio is near 1:1 and it is more close to stoichiometric ratio than some earlier reports [19,28]. The results of these measurements showed a good stoichiometry and homogeneity across the crystals. Slight Ga rich in composition is probably resulted from the loss of Se during synthesis and growth process because of its high vapor pressure.

### 3.2. Optical Measurements

**Figure 3:**
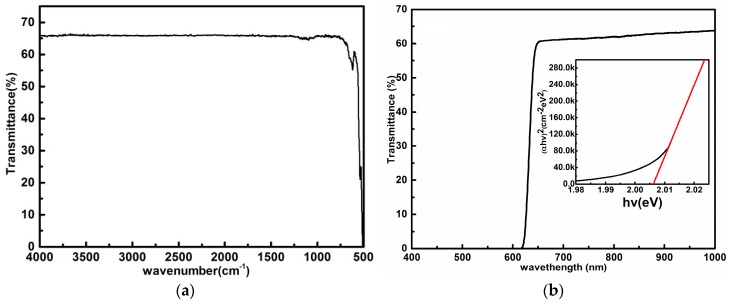
(**a**) Typical IR transmittance spectrum of a GaSe sample; and (**b**) the ultraviolet–visible–near IR spectrum of the GaSe sample.

The infrared-transmission spectrum is shown in Figure 3a, and the infrared transmittance is about 66% over the all range from 500 to 4000 cm^−1^, which is the highest ever reported for GaSe crystals grown by the Bridgman method. The optical absorption coefficient was calculated by the equation in [29,30]. The absorption coefficient is as low as 0.18–0.2 cm^−1^ in the range of 0.9–14 μm (see Supplementary Materials, Figure S2), indicating the perfect optical quality of GaSe sample. The ultraviolet–visible–near-IR spectrum of GaSe over the wavelength range from 200 nm to 2600 nm is shown in Figure 3b, from which the band gap is determined to be about 2.00 eV at room temperature.

**Figure 4:**
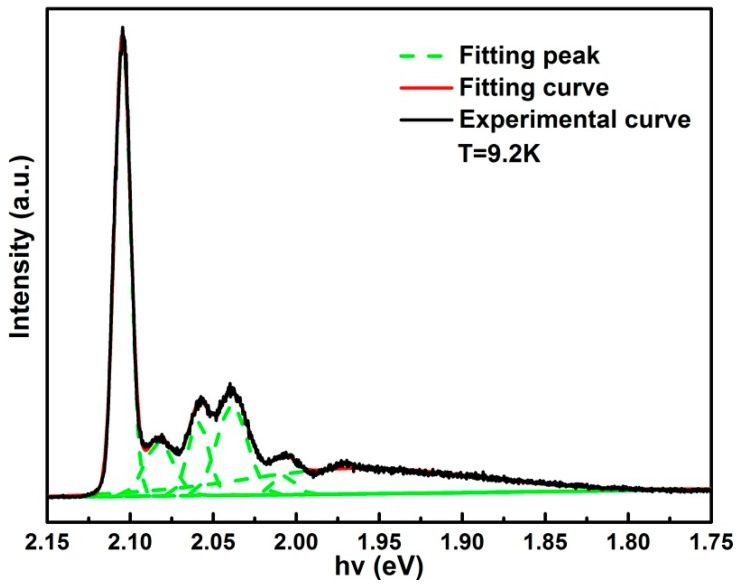
Typical PL spectrum of as-grown GaSe crystals at 9.2 K.

We also measured the photoluminescence (PL) spectrum of the as grown GaSe crystals at 9.2 K, which is shown in Figure 4. A dominant peak at 2.1046 eV was attributed to the direct free excitonic recombination. The strong and narrow peak with FWHM of 11 meV indicates excellent crystalline quality.

### 3.3. Electrical Measurements

Table 2 shows the Hall measurements of four GaSe samples taken from the tip to the tail of the ingot. All samples displayed p type conductivity and the hole concentration is about 10^15^ cm^−3^, with the mobility of ~20 cm^2^ V^−1^ s^−1^. The p-type conductivity may be contributed to the gallium vacancies (V_Ga_), interstitial selenium atoms (Se_I_), and gallium atoms on selenium sites (Ga_Se_) [31]. Since the composition measurement of the sample shows that Ga is slightly richer than Se, we conclude the p-type conductivity appeared, probably, due to the gallium atoms on selenium sites (Ga_Se_). The hole mobility is lower than the theoretical value of 50 cm^2^ V^−1^ s^−1^ and this may be contributed by the defects or traps in the surface of GaSe wafer. 

### 3.4. Characterization of the Few-Layered GaSe Photodetector

**Figure 5:**
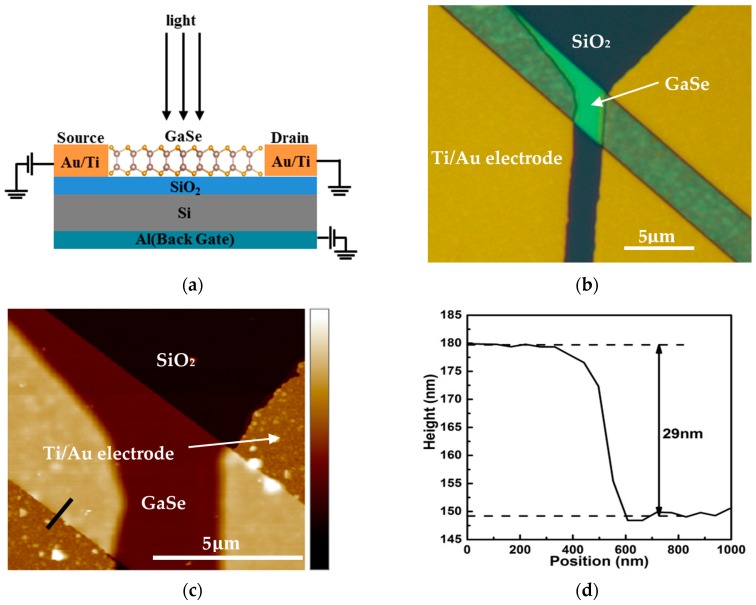
Few-layered GaSe photo-detector: (**a**) schematic diagram; (**b**) optical image; (**c**) AFM image; (**d**) AFM height profile along the black line.

Figure 5a shows the schematic diagram of the few-layered GaSe photodetector on the SiO_2_/Si substrate, and Figure 5b is the optical image of the fabricated few-layered GaSe photodetector. Figure 5c,d shows the Atomic Force Microscopy (AFM) image and thickness of this GaSe photodetector. The thickness along the black line is about ~29 nm. Considering that single layer GaSe has a thickness of 0.93 nm, the thickness corresponds to ~31 layers of GaSe. 

**Figure 6:**
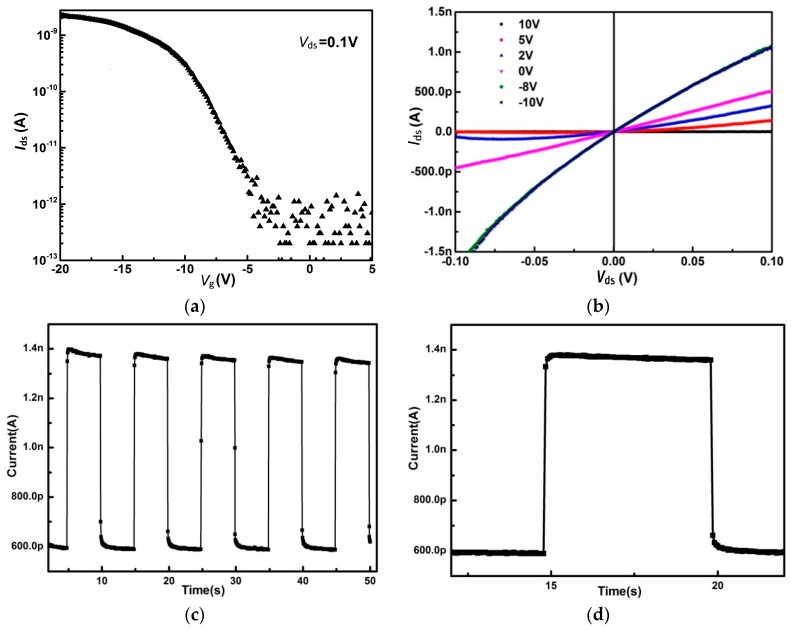
(**a**) FET transfer characteristics; (**b**) output characteristics; (**c**) photo-response under *V*_ds_ = −10 V; and (**d**) time resolved photoresponse in a single period.

Figure 6a,b shows the room temperature Field-Effect Transistor (FET) transfer characteristics and output characteristics of this device. The few-layer GaSe FET device shows a typical p-type conductive behavior, which is “off” under a positive gate voltage and “on” under a negative gate voltage. The on/off ratio is about 10^4^, which is comparable to earlier reports [13,14,19]. This high on/off ratio cannot be reached without the high-quality few-layered GaSe materials. The field-effect differential mobility of few-layered GaSe FET devices can be calculated by $\mu =g_{m}\times \left[L/\left(WC_{i}V_{ds}\right)\right]$ [10], where $g_{m}$ is the transconductance that equals to *dI_ds_*/*dV_gs_*, which can be obtained from the transfer curve, *L* and *W* are the length (7.84 μm) and width (3.75 μm) of the channel, and *C_i_* (6.9 × 10^−5^ F cm^−2^) is given by the equation *C_i_* = *ε*_0_*ε*_r_/*d*, where *ε*_r_ (3.9) and *d* (300 nm) are the dielectric constant and thickness of SiO_2_, respectively. The typical field-effect differential mobility for GaSe was calculated to be ≈0.4 cm^2^ V^−1^ s^−1^. Supposedly, the oxidation of the cleaved GaSe surface may be a source of this effect. At least, some comments could be given concerning this factor. The related information can be found elsewhere [32]. 

To further investigate the photoresponse of the device, a periodically-switched light with 520 nm was applied. The current exhibits a 1.5 nA light current and a 500 pA dark current, giving an *I*_light_/*I*_dark_ ratio of ~3, which is shown in Figure 6c. Figure 6d is the time resolved photoresponse in a single period, the response time is less than 60 ms with a sharp rise and decay. The switching between “on” and “off” states is very fast and stable, allowing the device to act as a high-quality photosensitive switch. This demonstrates that few-layered GaSe may be a suitable candidate for future electronic and optical devices.

## 4. Conclusions

In summary, high-quality GaSe ingots 22 mm in diameter and about 20 mm in length were grown by the vertical Bridgman method. The X-ray rocking curve is symmetric and the FWHM is 46 arcs. The IR transmittance is about 66% over the wavenumber range from 500 to 4000 cm^−1^. The PL spectrum shows a sharp near-band peak with a FWHM of 11 meV. All these results revealed that the as-grown GaSe crystals possessed high crystalline quality. The few-layered GaSe-based photo-detectors exhibit a 10^4^ on/off ratio, a field-effect differential mobility of 0.4 cm^2^ V^−1^ s^−1^, and have a fast response time less than 60 ms under light illumination.

## Figures and Tables

**Table 1:** The composition of GaSe measured by EPMA.

Sample	Se (Atom %)	Ga (Atom %)
1	49.5613	50.4387
2	49.5203	50.4797
3	49.6189	50.3811
4	49.5266	50.4734

**Table 2:** The transport properties of GaSe single crystals (T = 295 K).

Sample	Conductivity Type	Carrier Concentration (cm^−3^)	Resistivity (Ω·cm)	Mobility (cm^2^ V^−1^ s^−1^)
1	p	2.9894 × 10^15^	1.129546 × 10^2^	1.8484 × 10^1^
2	p	1.4701 × 10^15^	2.332230 × 10^2^	1.8204 × 10^1^
3	p	1.6830 × 10^15^	1.621991 × 10^2^	2.3492 × 10^1^

## Supplementary Materials

The following are available online at http://www.mdpi.com/1996-1944/11/2/186/s1, Figure S1. SEM pictures of GaSe (001) face; Figure S2. Calculated absorption coefficient of GaSe crystals.
