# Supplementary material for: High-Quality GaSe Single Crystal Grown by the Bridgman Method
Source: Materials (Basel). 2018 Jan 24;11(2):186. doi: 10.3390/ma11020186 (PMC5848883; doi:10.3390/ma11020186)
Supplement: Supplementary file 1 [file materials-11-00186-s001.pdf]

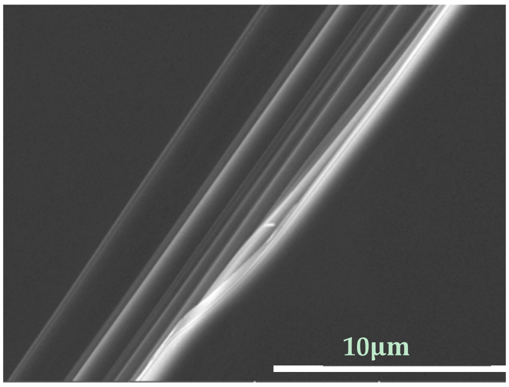

Figure S1. SEM pictures of GaSe (001) face

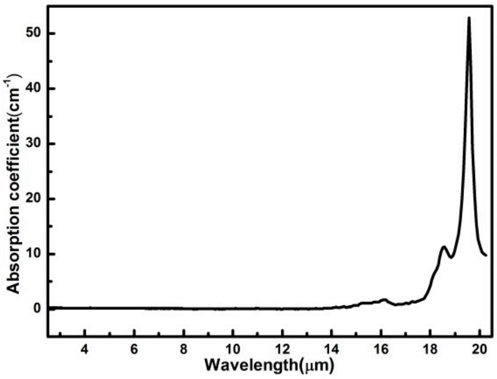

Figure S2. Calculated absorption coefficient of GaSe crystals
